# The prognostic value of growth pattern-based grading for mucinous ovarian carcinoma (MOC): a systematic review and meta-analysis

**DOI:** 10.3389/fonc.2025.1541572

**Published:** 2025-03-31

**Authors:** Mengmeng Chen, Ling Han, Yisi Wang, Qi Qiu, Yali Chen, Ai Zheng

**Affiliations:** ^1^ Department of Obstetrics and Gynecology, West China Second University Hospital, Sichuan University, Sichuan, China; ^2^ Key Laboratory of Birth Defects and Related Diseases of Women and Children (Sichuan University), Ministry of Education, Sichuan, China

**Keywords:** mucinous ovarian carcinoma, pattern-based grading, expansile, infiltrative, prognosis, meta-analysis

## Abstract

**Objective:**

To investigate the prognostic significance of expansile and infiltrative growth patterns in mucinous ovarian carcinoma (MOC).

**Methods:**

A systematic search was conducted in the PubMed, Embase, and Web of Science databases for studies published between January 1, 2010, and September 6, 2024, examining the correlation between expansile and infiltrative tumor growth patterns and prognosis in MOC. Subgroup analyses were performed for mortality, recurrence, and FIGO stage I based on tumor subtype. The Chi-square test was used to evaluate the distribution of expansile and infiltrative tumors across FIGO stages I-IV.

**Results:**

Twelve eligible studies, comprising a total of 1185 patients, were included in this systematic review and meta-analysis. The combined death rate in the expansile and infiltrative MOC was 10.5% (95%CI: 6.2-15.7) and 31.1% (95%CI: 14.1-50.9). The combined recurrence rate in the expansile and infiltrative MOC was 6.9% (95%CI: 3.1-11.9) and 24.5% (95%CI: 14.3-36.2). The combined International Federation of Gynecology and Obstetrics (FIGO) I rate in the expansile and infiltrative MOC was 89.8% (95%CI: 84.9-94.0) and 56.2% (95%CI: 41.5-70.4). A significant association was found between tumor type and FIGO stage (χ² (3) = 110.92, p < 0.00001).

**Conclusion:**

Expansile MOC predicts better outcomes, while infiltrative MOC is linked to advanced stages and poorer prognosis. Complete surgical staging is crucial for infiltrative MOC but optional for early-stage expansile MOC. Early-stage patients should consider fertility-sparing surgery, timely conception, and close recurrence monitoring.

## 1 Introduction

Ovarian cancer is the second most common gynecological malignancy (1). Among its various subtypes, high-grade serous ovarian carcinoma (HGSC) is the most prevalent histological subtype, while mucinous ovarian carcinoma (MOC) is quite rare, constituting approximately 3% to 11% of ovarian cancers (2, 3). MOC is recognized as a distinct entity from other epithelial ovarian cancers (EOCs), exhibiting a unique natural history, molecular profile, chemo-sensitivity, and prognosis compared to HGSC. Notably, MOC is the most common subtype in women under 40 (4), with tobacco smoking identified as the only significant risk factor (5). While most HGSC cases are diagnosed at advanced stages, 80% of MOC cases are identified at stage I (6). Early-stage MOC typically exhibits a better prognosis, however, advanced cases face poorer outcomes, primarily due to a limited response to platinum-based chemotherapy compared to HGSC (7, 8).

Histological grading systems, such as the International Federation of Gynecology and Obstetrics (FIGO) and Silverberg grading systems, have been studied in relation to the ovarian cancer patient prognosis, including MOC (9, 10). As yet, these grading systems alone are insufficient for predicting the clinical course of MOC, unlike their application for other ovarian carcinoma subtypes (11). In 2014, in order to standardize the pathological reporting of gynecological tumors, World Health Organization (WHO) guidelines proposed classifying the mucinous cancers in these two groups based on their growth patterns, calling them expansileand infiltrative-type tumors (12), which was also entered in the newest version CAP protocols (13). However, there is controversy over the treatment of this histological groups using different compasses. Guidelines from the European Society for Medical Oncology and the European Society of Gynecological Oncology (ESMO-ESGO) emphasize the importance of adjuvant chemotherapy for stage IB-IC infiltrative MOC. Even for stage IA, adjuvant chemotherapy may be considered for patients with infiltrative patterns, whereas it is not deemed necessary for stage IA expansile MOC (14, 15). Conversely, the National Comprehensive Cancer Network (NCCN) guidelines do not recommend differentiating histologic subtypes when treating patients with MOC. Instead, they advise administering adjuvant chemotherapy for stage IC or higher MOC, while treatment can be avoided for stage IA-IB, similar to other EOCs (16).

Therefore, we conducted a meta-analysis and systematic review aimed at assessing the prognostic significance of the expansile and infiltrative growth patterns in MOC. This study seeks to provide clearer guidance for the treatment of MOC and improve clinical management and outcomes for patients.

## 2 Methods

### 2.1 Protocol registration

This meta-analysis was conducted in accordance with the Preferred Reporting Items for Systematic Reviews and Meta-Analyses (PRISMA) guidelines (17). Prior to data extraction, the review was registered with the International Prospective Register of Systematic Reviews (PROSPERO) under registration number CRD42024585615.

### 2.2 Eligibility criteria and exclusion criteria

#### 2.2.1 Eligibility criteria

To be eligible, we aimed for the following inclusion criteria: 1) The study design is a retrospective or prospective study design;2) Included cases need to be classified by expansile or infiltration subtype, and need to be confirmed the diagnosis of MOC;3) Included articles assess at least one of the following parameters: death, recurrence, FIGO I or FIGO stage.

#### 2.2.2 Exclusion criteria

We excluded studies with the following exclusion criteria:1) Reviews, letters, case reports or editorial comments;2) Studies without full text, insufficient data or low-quality scores based on Newcastle- Ottawa Scale (NOS) (18);3) Republished literature or repetitive studies.

### 2.3 Search strategy

Two researchers (MMC and YSW) conducted a comprehensive search in electronic databases of PubMed, Embase, and Web of Science for relevant researches, published for from January 1, 2010 to September 6, 2024.

The following search terms were used to identify relevant studies on ovarian cancer: “Carcinoma, Ovarian Epithelial”, “Epithelial Carcinoma, Ovarian”, “Ovarian Epithelial Carcinomas”, whereas the following terms were used to identify relevant studies on expansile and infiltrative: “expansile”, “infiltrative”.

Two researchers (LH and YLC) thoroughly reviewed the reference lists of all included articles to identify any potentially missing studies or unpublished data. In cases where multiple studies analyzed overlapping patient populations, the most recent or comprehensive results were selected. Following the initial screening, the full texts of all potential articles were independently reviewed by two researchers (QQ and MMC) for further evaluation. Any disagreements were resolved through discussion with AZ.

### 2.4 Data extraction

Data were independently extracted by two investigators (QQ and YSW), with any disagreements resolved through discussion with AZ. The extracted data included author, publication date, country, number of cases, growth patterns (expansile and infiltrative), oncological outcomes (death, recurrence), and pathological characteristics (FIGO stage). Attempts were made to obtain missing data by contacting the authors via email; however, no responses were received.

#### 2.4.1 Expansile and infiltrative pattern

In expansile tumor, the tumor consists of a confluent glandular growth pattern with minimal to no stromal invasion. In contrast, infiltrative tumor shows malignant cell clusters with destructive stromal invasion (12).

#### 2.4.2 Oncological outcomes

Death was calculated from the data from surgery to either the last follow-up or the data of death. Recurrence refers as either pathologic proof of cancer or an imaging study showing the regrowth of the tumor, whether it is confined to the pelvic region or outside of it.

#### 2.4.3 Pathological features

For mucinous ovarian carcinoma, Stage I means tumor confined to the ovaries, Stage II means tumor involves one or both ovaries and extends to other pelvic tissues, such as the uterus or fallopian tubes. Stage III means tumor is present in one or both ovaries and has spread to the peritoneum outside the pelvis or to regional lymph nodes. Stage IV means tumor has spread beyond the peritoneum to distant organs, such as the liver or lungs.

### 2.5 Quality assessment

Two reviewers (MMC and YSW) independently assessed the quality of the included studies, with disagreements resolved through discussion. The quality of each study was evaluated using the Newcastle-Ottawa Scale (NOS), which assesses three categories: case selection, comparability between groups, and outcome assessment. The total NOS score ranges from 0 to 9 points, and studies with a score of ≥6 were considered high-quality and included in our analysis.

### 2.6 Statistical analysis

Meta-analysis was performed by using STATA 15.0 software. Subgroup analyses were based on expansile and infiltrative pattern, and heterogeneity was determined using orthorhombic test and I^2^ statistic. If there was significant heterogeneity (p-value <0.05 or I^2^ >50%), a random-effects model was used. Otherwise, a fixed-effect model was used (19). Additionally, a Chi-Square Test was performed to evaluate whether there were statistical differences in the distribution of expansile tumors and infiltrative tumors across stages I, II, III, and IV. Sensitivity analysis to determine the robustness and stability of the results, calculating the herogeneity in each situation in which a single study was removed in turn in noder to evaluate the effect of a single study on the overall outcome. Risk of publication was assessed by visual inspeciton of Begg’s funnel plot.

## 3 Result

### 3.1 Study selection and characteristics

**Figure 1:**
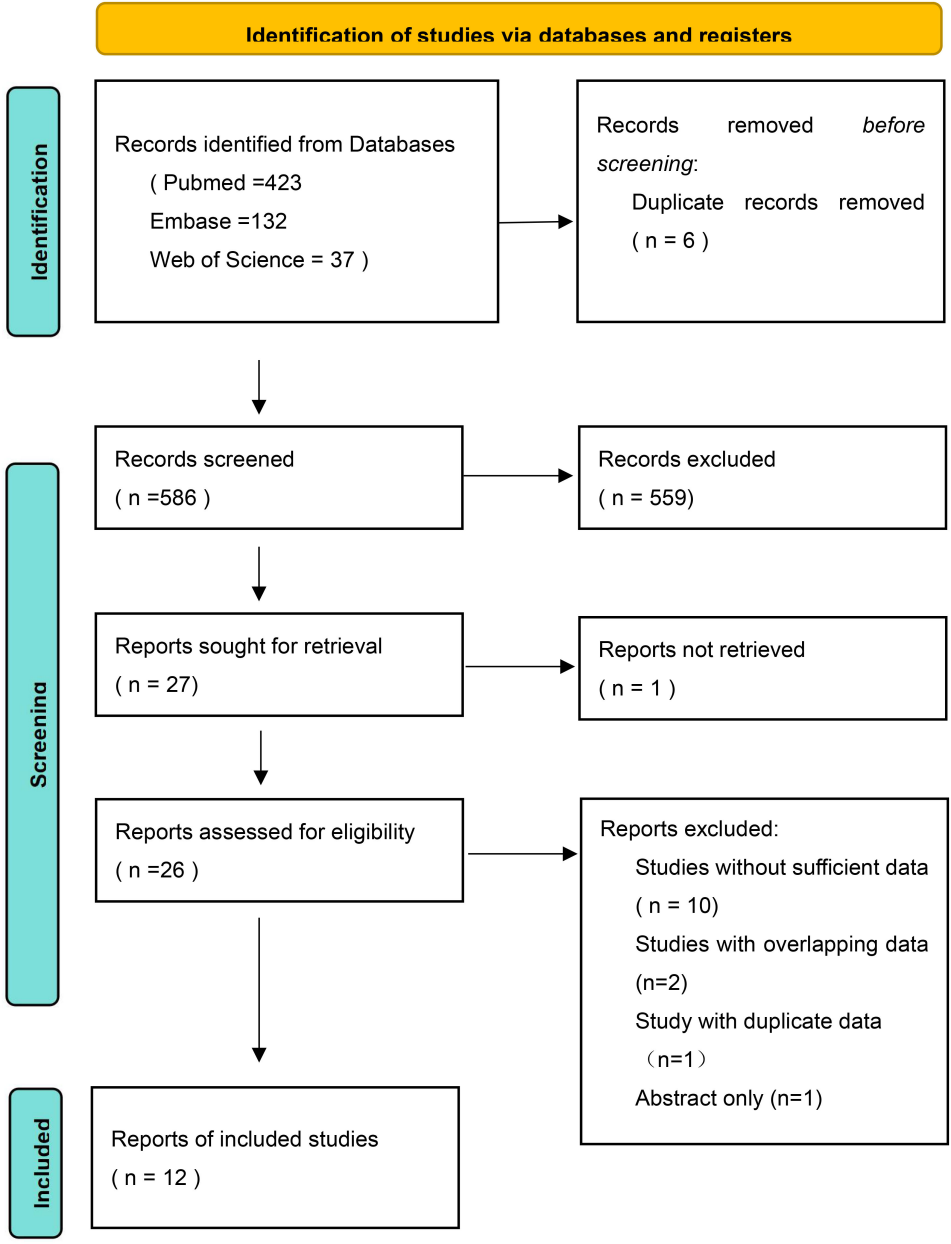
Flow diagram of the included studies.

The initial search retrieved a total of 592 relevant studies from three databases (PubMed = 423, Embase = 132, Web of Science = 37). After removing duplicates and screening titles and abstracts, 27 studies remained. Following a full-text evaluation, 15 studies were excluded. Ultimately, 12 studies, including 1185 patients, met the inclusion criteria and were included in this meta-analysis. A flowchart of the selection process is provided in **Figure 1**.

All included studies were retrospective and received seven or more stars based on the NOS criteria. The quality assessments of these studies are presented in **Table 1**, while the general characteristics of the studies included in this meta-analysis are summarized in **Table 2**.

**Table 1:** Quality assessment of included studies.

Study	Selection				Comparability			Outcome		Total
	Representativeness	Selection of non-exposed	Ascertainment of exposure	Outcome not present at start	Comparability on most important factors	Comparability on other risk factors	Assessment of outcome	Long enough follow-up (median>=5 year)	Adequacy (completeness of follow-up)	
Gouy S (20)	✓	✓	✓	✓	✓	×	✓	✓	✓	8
Lim H (21)	✓	✓	✓	✓	✓	×	✓	×	✓	7
Hada T (22)	✓	✓	✓	✓	✓	×	✓	×	✓	7
Tabrizi AD (23)	✓	✓	✓	✓	✓	×	✓	×	✓	7
Sotiropoulou M (24)	✓	✓	✓	✓	✓	×	✓	✓	✓	8
Algera MD (25)	✓	✓	✓	✓	✓	×	✓	×	✓	7
Meagher N (26)	✓	✓	✓	✓	✓	×	✓	×	✓	7
Huin M (27)	✓	✓	✓	✓	✓	✓	✓	✓	✓	8
Muyldermans K (9)	✓	✓	✓	✓	✓	×	✓	✓	✓	8
Hada T (28)	✓	✓	✓	✓	✓	×	✓	×	✓	7
Nistor S (29)	✓	✓	✓	✓	✓	×	✓	×	✓	7
Köbel M (30)	✓	✓	✓	✓	✓	×	✓	×	✓	7

“√” indicates that the criteria are met, while “×” indicates that the criteria are not met.

**Table 2:** The basic characteristics of included studies.

First author	Publish year	Study period	Region	Study design	Cases	Follow up	Quality
Gouy S (20)	2018	1976-2016	France	R	64	62m	8
Lim H (21)	2023	2003-2021	Korea	R	113	55m	7
Hada T (22)	2022	1984-2019	Japan	R	52	54m	7
Tabrizi AD (23)	2010	1984-2000	Iran	R	31	NM	7
Sotiropoulou M (24)	2013	1998-2008	Greece	R	42	6y	8
Algera MD (25)	2024	2015-2020	Netherlands	R	409	999d	7
Meagher N (26)	2021	NM	Australia	R	133	2y	7
Huin M (27)	2022	2001-2019	France	R	94	5y	8
Muyldermans K (9)	2013	1993-2011	Belgium	R	44	63m	8
Hada T (28)	2021	1984-2018	Japan	R	49	NM	7
Nistor S (29)	2023	2010-2022	UK	R	33	37m	7
Köbel M (30)	2024	NM	Canada	R	121	NM	7

“d” means day, “m” means month and “y” means year. “R” means retrospective. “NM” means not mentioned.

### 3.2 Subgroup analysis based on expansile and infiltration tumors.

#### 3.2.1 Death

**Figure 2:**
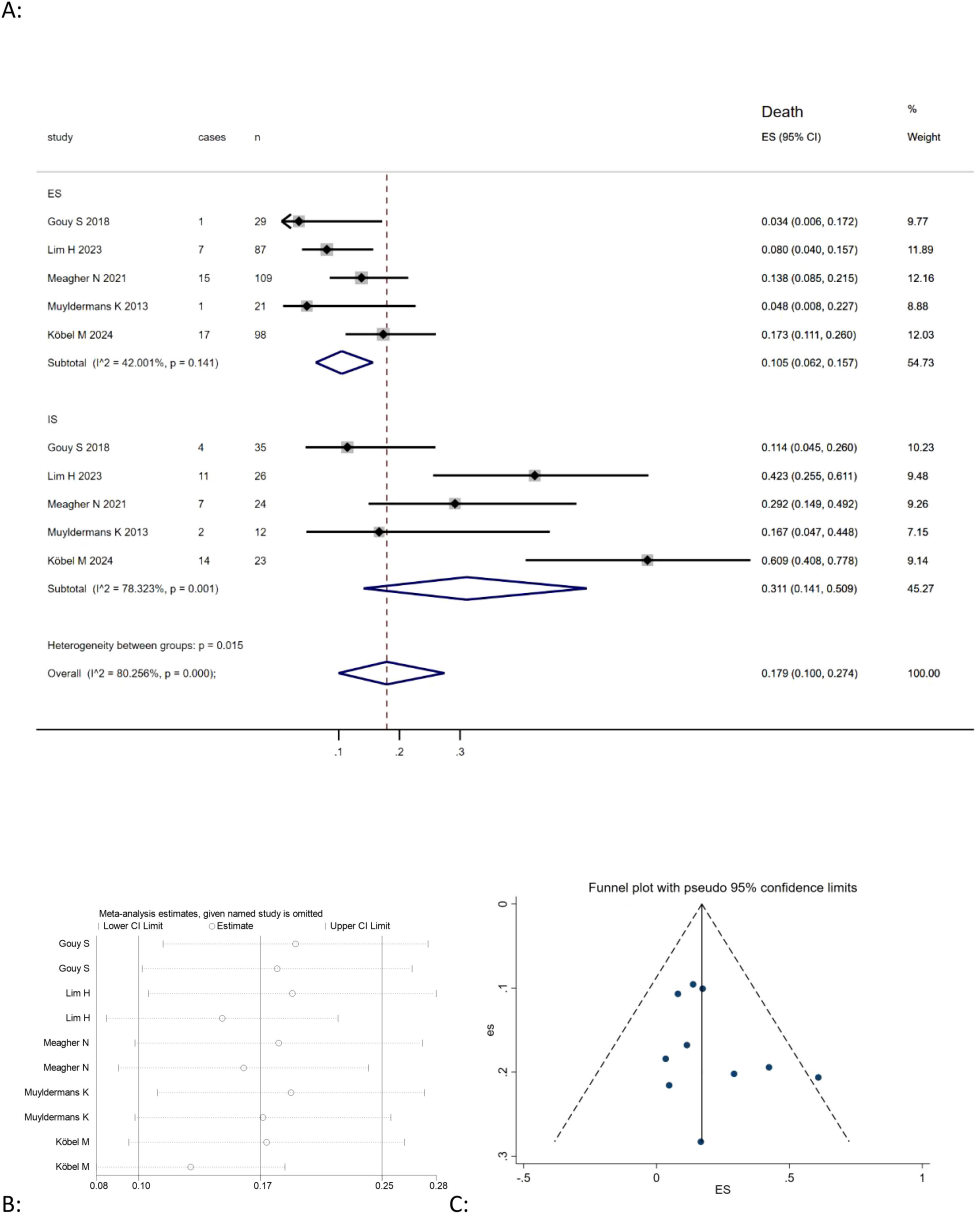
**(A)** Forest plots showing the relationship between infiltrative subtype, expansile subtype, and death rate in MOC; **(B)** sentivity analysis to evaluate robustness and **(C)** funnel plots show publication bias by visual inspection.

This meta-analysis of five studies (9, 20, 21, 26, 30) showed that the combined death rate of mucinous ovarian carcinoma was positively correlated with expansile patter (Effect Size=0.105, 95%CI=0.062-0.157, I^2^ = 42.001%, n=5), while no significant correlation for infiltrative pattern (Effect Size=0.311, 95%CI=0.141-0.509,I^2^ = 78.323%, n=5) **Figure 2A**. However, the results also indicated high heterogeneity among the studies (I^2^ = 80.256%, p<0.05). In order to assess the stability of the model, sensitivity analysis was conducted by excluding each individual study and calculating new effect size. The results showed that the effect size were relatively stable, as illustrated in **Figure 2B**.

#### 3.2.2 Recurrence

**Figure 3:**
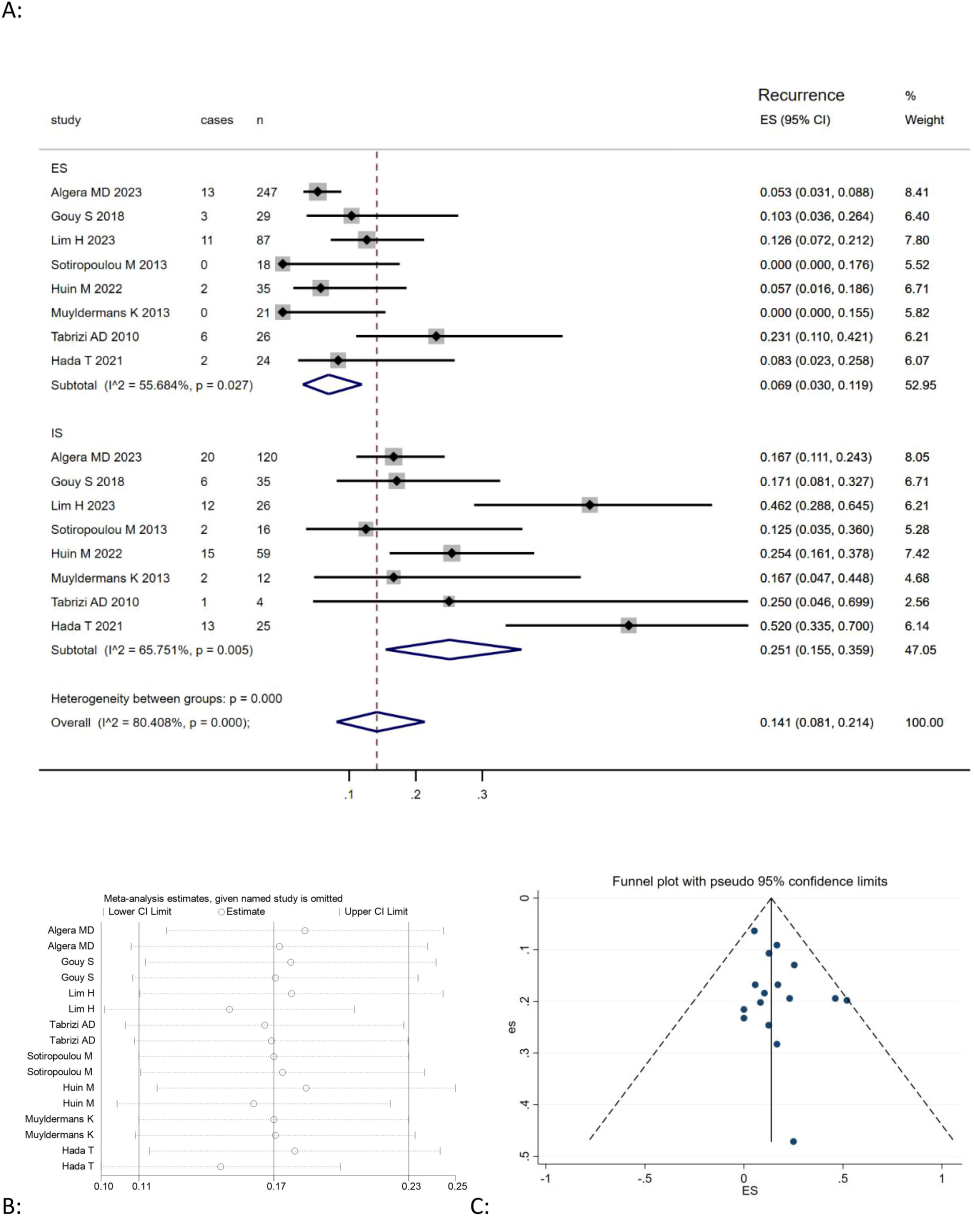
**(A)** Forest plots showing the relationship between death rate and infiltrative subtype, expansile subtype; **(B)** sentivity analysis to evaluate robustness and **(C)** funnel plots show publication bias by visual inspection.

This meta-analysis of eight studies (9, 20, 21, 23–25, 27, 28) showed that the combined recurrence of mucinous ovarian carcinoma was positively correlated with expansile pattern (Effect Size=0.069, 95%CI=0.031-0.119, I^2^ = 55.150%, n=8), negatively correlated with infiltrative pattern (Effect Size=0.245, 95%CI=0.143-0.362,I^2^ = 79.797%, n=8) **Figure 3A**. The findings also revealed significant heterogeneity among the studies (I^2^ = 80.408%, p<0.05). A sensitivity analysis was performed by omitting each study individually and recalculating the effect size to evaluate model stability. The results indicated that the effect sizes remained fairly stable, as shown in **Figure 3B**.

#### 3.3.3 FIGO I and FIGO stage

**Figure 4:**
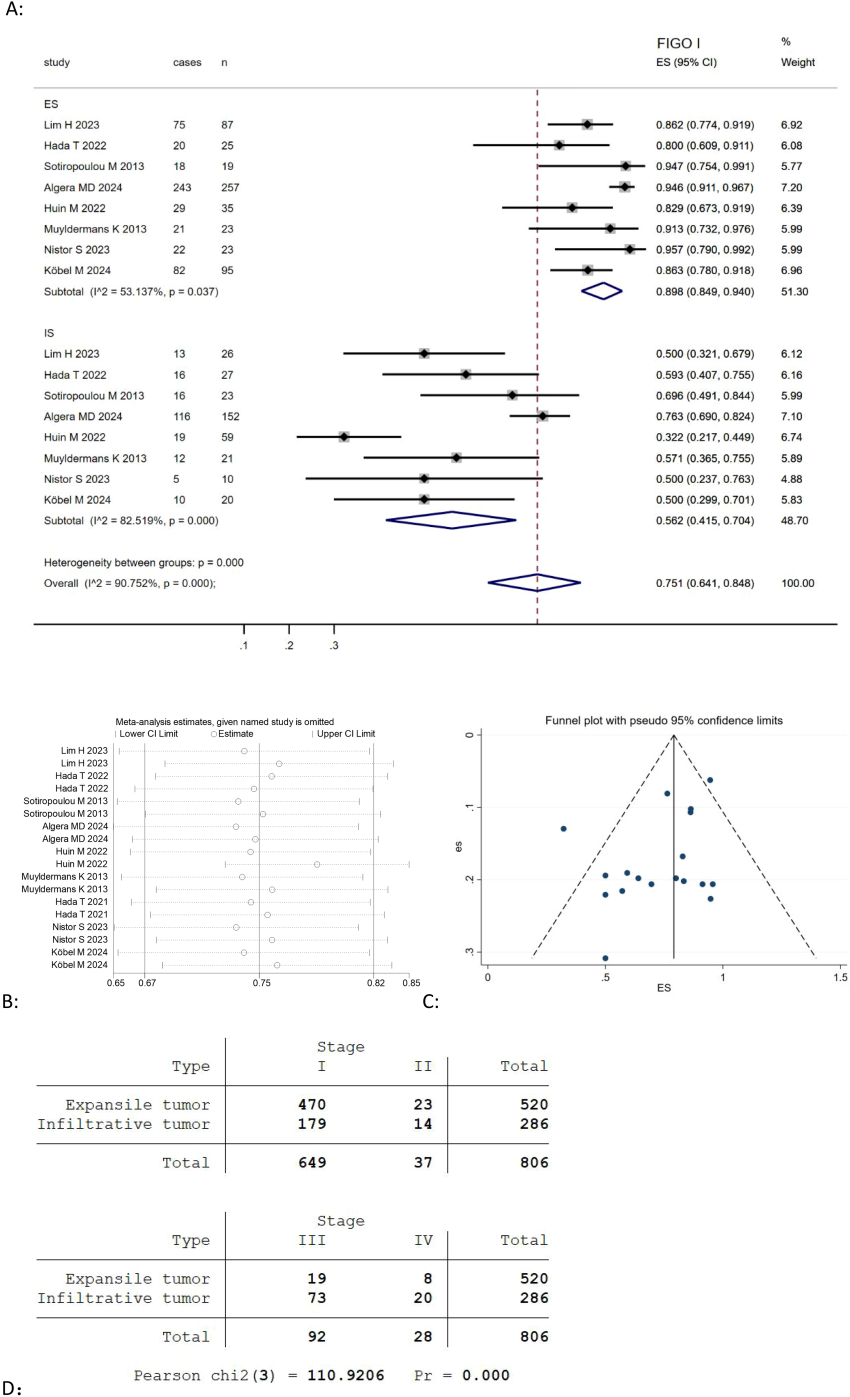
**(A)** Forest plots showing the relationship between FIGO I rate and infiltrative subtype, expansile subtype; **(B)** sentivity analysis to evaluate robustness and **(C)** funnel plots show publication bias by visual inspection; **(D)** Cross-tabulation of the distribution of expansile and infiltrative MOC by FIGO stage (I-IV).

Given that most MOC cases are diagnosed at an early stage, we selected FIGO stage I as one of the key pathological features in our study and found eight studies (**Table 3**) (9, 21, 22, 24, 25, 27, 29, 30) reported the association between the expansile and infiltrative pattern for mucinous ovarian carcinoma and FIGO I stage. The result revealed that the combined FIGO I stage rate of mucinous ovarian carcinoma was positively correlated with expansile pattern (Effect Size=0.898, 95%CI=0.849-0.940, I^2^ = 53.137%, n=8), negatively correlated with infiltrative pattern (Effect Size=0.562, 95%CI=0.415-0.704, I^2^ = 82.519%, n=8) **Figure 4A**. Moreover, the results highlighted considerable heterogeneity across the studies (I^2^ = 90.752%, p<0.05). To evaluate the robustness of the model, a sensitivity analysis was carried out by removing each study one at a time and recomputing the effect size. The findings suggested that the effect sizes were largely consistent, as depicted in **Figure 4B**.

**Table 3:** Distribution of expansile and infiltrative MOC patients across FIGO stages I-IV in various studies.

	Expansile Tumor Stage				Infiltrative Tumor Stage			
	I	II	III	IV	I	II	III	IV
Algera MD (25)	243	6	7	1	116	7	23	2
Lim H (21)	75	3	5	4	13	0	8	5
Hada T (22)	20	2	1	2	16	1	7	3
Huin M (27)	28	1	3	0	19	0	27	9
Nistor S (29)	22	2	0	–	5	3	2	–
Köbel M (30)	82	9	3	1	10	3	6	1

Besides, we use the Pearson Chi-Square test to evaluate the distribution of FIGO stages I, II, III, IV among expansile and infiltrative tumors, and found there was a highly significant association between tumor type and FIGO staging (Pearson chi2(3) = 110.9206, p <0.00001) **Figure 4D**.

#### 3.3.4 Publication bias

Publication bias was investigated by Begg’s funnel plots. Visual inspection of the Begger’s funnel plot was almost symmetrical, as depicted in **Figures 2C**, **3C**, **4C**, suggesting no obvious evidence of publication bias.

## 4 Discussion

This meta-analysis revealed that mucinous ovarian carcinoma with expansile-pattern tumors, typically observed in early-stage, tend to have a better oncological prognosis. In contrast, infiltrative-pattern tumors are commonly associated with advanced stages and are linked to poorer outcomes.

Our study indicated that patients with expansile pattern tumors have lower death rate, recurrence rate and a higher proportion of FIGO stage I compared to those with infiltrative tumors. A study conducted by Taira Hada et al. (22) showed that MOC with expansile invasion was a better prognostic factor for progression-free-survival and overall survival than HGSC at all stage. Besides, Taira Hada et al. (31) also conducted a study, and found there was no statistically significant differences in the recurrence rate and prognosis of MOC with expansile and mucinous borderline tumors, it might be possible that expansile MOC biologically behave more like mucinous borderline tumors. These studies suggest that expansile MOC is not an aggressive subtype, leading many researchers to question whether comprehensive staging surgery is necessary for early-stage expansile tumors. Marc D et al. (25) conducted a study of peritoneal staging in clinical early-stage MOC, found limited benefit for routinely performing peritoneal and lymph node staging procedures in patients with expansile tumors, because recurrences, overall survival and recurrence free survival were similar across the different extent of surgical staging groups. In another study (15), researchers concluded that peritoneal metastases are rare in expansile MOC, more than 90% of patients have early-stage disease. Gouy S et al. (32) describes no lymph node involvement in expansile tumors, while one patient upstaged after surgical staging, based on positive peritoneal cytology (3.4%, one out of 29 patients). In conclusion, expansile is a less aggressive pattern. For patients with early-stage expansile MOC, it may be considered safe to forgo additional staging surgery and lymph node sampling following the initial bilateral salpingo-oophorectomy and hysterectomy. Nevertheless, further data is needed to validate this observation and ensure that patient outcomes are not compromised.

In contrast, infiltrative tumors are typically associated with more advanced stages and higher recurrence rates than expansile tumors. Gouy S et al. (20) found lethal recurrences were observed mainly in infiltrative type. Taira Hada et al. (22) reported that univariate analysis showed that MOC with infiltrative invasion at FIGO stages II to IV had worse progression free survival and overall survival than HGSC. Due to the high recurrence rate, it might be considered adjuvant treatment for infiltrative tumor, even in early-stage. According to Lim H et al. (21), one-third of patients who received lymphadenectomy had lymph node involvement. Gouy S et al. (32) investigated 31 infiltrative MOC underwent staging operations and found four patients had nodal involvement. Hence, we suggest lymphadenectomy must be considered during staging operations in patients with infiltrative tumor. Algera MD et al. (15) concluded that upstaging clinical early-stage infiltrative MOC based on positive cytology, peritoneum and omentum metastases occurred in 10.3% of the patients. Besides, Marc D et al. (25) conducted a study of peritoneal staging in clinical early-stage MOC, found that in the infiltrative cohort, overall survival was better for patients undergoing full staging compared with those undergoing fertility sparing or partial staging, patients undergoing fertility-sparing staging for infiltrative tumors experienced significantly more recurrences. In conclusion, patients diagnosed with infiltrative mucinous ovarian carcinoma (MOC) should undergo a thorough surgical staging process. This process should include peritoneal staging, which involves omentectomy, the collection of peritoneal washings, and the acquisition of biopsies, along with pelvic and para-aortic lymph node sampling. Given the potential aggressiveness of this type of cancer, adjuvant treatment should be considered even for tumors identified at an early stage.

In recent years, research on the molecular characteristics of mucinous ovarian cancer (MOC) has increased, providing new insights into its invasion patterns and prognosis. A study found that mucinous ovarian cancer (MOC) with infiltrative invasion was more often positive for CK5/6, CD24, and EGFR, suggesting that these markers may be linked to the aggressive features of this invasion pattern (28). In contrast, expansile invasion showed a higher prevalence of HER2 overexpression/amplification and less frequent HER2 mutation compared to infiltrative MOC, although this difference was not statistically significant (33). Additionally, PAX8 expression was more commonly associated with expansile invasion, but the difference was not statistically significant (75% vs 37.5%, p=0.99) (29). Overall, the existing data are limited, highlighting the need for further research to integrate molecular data with histological classification for a comprehensive understanding of MOC prognosis.

Fertility-sparing surgery (FSS) is a common topic of discussion because patients diagnosed with MOC are often younger. In recent years, preserving fertility becomes a significant concern in treatment planning, and several studies have focused on the outcomes of fertility-sparing surgery in patients with early-stage MOC. Gouy S et al. (34) conducted a study and emphasized that FFS should be considered for early-stage MOC regardless of its subtype. Similarly, Yoshihara M et al. (35) found patients with stage I MOC underwent uterus preserving surgery was not associated with decreased survival. On the other hand, Hyunji Lim et al. (21) found infiltrative tumors showed no statistical significance with worse survival, but patients in the infiltrative tumors group who underwent FSS demonstrated a 5-year progression free survival rate of 83.3%, significantly lower than patients without fertility preservation. This suggests that adjuvant chemotherapy should be considered for patients with stage I disease who have undergone FSS, particularly if the histologic subtype is infiltrative. Bentivegna et al. Reported the long-term outcome of 280 MOC patients treated with FFS, the recurrence rate was 6,8% (36). Additional, Wei Lin et al. (37) noted no significant difference in disease-free survival between the FSS and radical surgery groups in patients with stage IA and IC disease, though the FSS group did show a trend toward poorer disease-free survival compared to those who underwent radical surgery. Besides, they found that, among 23 patients diagnosed with early-stage mucinous ovarian carcinoma who underwent fertility-sparing surgery (FSS) and attempted to conceive, 21 (91.3%) successfully achieved 27 pregnancies. These included 26 spontaneous pregnancies and one pregnancy resulting from assisted reproductive technology. However, there is a lack of data on the recurrence rates associated with FSS, highlighting the need for further research in this area. More studies should be conducted to better understand the long-term outcomes and potential risks of recurrence following FSS in patients with mucinous ovarian carcinoma. But we strongly recommend FSS for patients with early-stage MOC, irrespective of the tumor subtype. This approach aims to preserve fertility while effectively treating the cancer. Following treatment, these patients should be encouraged to attempt conception as soon as they are medically cleared and should engage in regular follow-up to monitor for any signs of relapse.

This meta-analysis is the first to evaluate the relationship between growth patterns and prognosis in MOC, but it has limitations. One of the most obvious limitation is the high heterogeneity among the results, although we did sensitivity analysis to explain its robustness, we are currently unable to perform a more thorough investigation into the sources of heterogeneity due to incomplete data. All included studies were retrospective, which may affect the results. Additionally, only English language studies were considered, potentially introducing language bias. The subgroup analysis did not show a significant link between infiltrative patterns and death rate due to limited data. Despite these limitations, the study offers initial insights into the prognostic importance of growth patterns in MOC and suggests areas for future research, calling for more studies, including those with negative findings, to support these conclusions.

## 5 Conclusion

Our study found that expansile MOC generally has better outcomes, while infiltrative MOC is associated with poorer prognosis and advanced stages. Full surgical staging is recommended for infiltrative MOC, but may be omitted for early-stage expansile MOC. Fertility-sparing surgery is advised for early-stage patients, with early conception and close monitoring.

## Data Availability

The original contributions presented in the study are included in the article/Supplementary Material. Further inquiries can be directed to the corresponding author/s.
